# Metabolic Detoxification of Glucose and 4-Hydroxynonenal in Human Neuroblastoma Cell Models

**DOI:** 10.3390/antiox15030298

**Published:** 2026-02-27

**Authors:** Martina Avanatti, Gemma Sardelli, Rossella Mosca, Francesco Balestri, Giovanni Signore, Francesca Felice, Antonella Del Corso, Roberta Moschini

**Affiliations:** 1 Biochemistry Unit, University of Pisa, Via San Zeno 51, 56123 Pisa, Italy; m.avanatti@studenti.unipi.it (M.A.); gemma.sardelli@phd.unipi.it (G.S.); r.mosca@studenti.unipi.it (R.M.); francesco.balestri@unipi.it (F.B.); giovanni.signore@unipi.it (G.S.); antonella.delcorso@unipi.it (A.D.C.); roberta.moschini@unipi.it (R.M.); 2 Interdepartmental Research Center Nutrafood “Nutraceuticals and Food for Health”, University of Pisa, 56123 Pisa, Italy

**Keywords:** neuroblastoma, hyperglycemia, oxidative stress, aldose reductase, carbonyl reductase 1

## Abstract

Background: Neuroblastoma (NB) progression is influenced by metabolic and redox adaptations. The polyol pathway, driven by aldose reductase (AKR1B1) and sorbitol dehydrogenase (SORD), is activated in hyperglycemic conditions, while detoxification of lipid peroxidation products such as 4-hydroxynonenal (4-HNE) involves carbonyl reductase 1 (CBR1) and AKR1B1. A systematic characterization of these enzymes under distinct metabolic and oxidative challenges in NB is currently lacking. Methods: Human neuroblastoma LAN-5 and SH-SY5Y cells were exposed to hyperglycemic medium to assess polyol pathway regulation, and to exogenous 4-HNE to model aldehyde-induced oxidative stress. Protein expression and enzyme activities were quantified. Cells were treated with Sorbinil or rutin during stress exposure, and viability was analyzed in 2D and 3D models. Results: Hyperglycemia increased AKR1B1 activity and sorbitol accumulation, indicating polyol pathway activation in NB cells. Both NB cell lines displayed an incomplete HNE-detoxifying enzyme profile, with absence of ALDH1A1 and AKR1C3 expression. Exposure to 4-HNE reduced NB cell viability both in 2D and 3D models. Pharmacological inhibition of AKR1B1, but not of CBR1, exacerbated 4-HNE-mediated cytotoxicity. Conclusions: While hyperglycemia stimulates the polyol pathway, aldehyde detoxification by AKR1B1 supports resistance to 4-HNE toxicity, demonstrating that AKR1B1 activity is essential to counteract HNE toxicity, and its impairment may increase the susceptibility of NB cells to oxidative damage.

## 1. Introduction

Neuroblastoma (NB), the most common extracranial solid tumor of childhood, is characterized by marked heterogeneity and a strong metabolic component that influences tumor aggressiveness and therapeutic response [1]. Oxidative and metabolic stress are common features of both cancer and diabetic complications, often involving similar detoxification systems. Increasing evidence highlights the contribution of metabolic alterations and redox imbalance in NB progression. In particular, hyperglycemia, a common metabolic stressor in diabetes and cancer, has been shown to exacerbate oxidative stress and promote pro-inflammatory signaling, thereby contributing to tumor aggressiveness [2]. NB tumors exhibit high glucose uptake and increased lactic acid production, representative of the metabolic switch from OXPHOS to glycolysis [3]. Consistently, as reported by de Grauw et al. [4], hyperglycemia resulted in the initial presenting symptom of a metabolically active NB in an 11-month-old girl. Moreover, NB cell death is dose-dependently influenced by increasing glucose concentrations [5].

A key pathway activated under hyperglycemic conditions is the polyol pathway, in which aldose reductase (AKR1B1) catalyzes the reduction of glucose to sorbitol, followed by oxidation to fructose by sorbitol dehydrogenase (SORD) [6]. However, the dysregulation of this pathway and its contribution to NB adaptation under high-glucose conditions remains unclear.

NB cells can adapt to intrinsic oxidative stress by upregulating their antioxidant capacity, which on the one hand enables the cancer cells to survive under elevated ROS levels, and on the other hand contributes to resistance against multiple anticancer agents and radiation therapy [7]. In this context, it has been observed that lipid peroxidation plays an important role in the development of primary and metastatic brain tumors [8]. Among the major end-products of lipid peroxidation are reactive aldehydes, such as 4-hydroxynonenal (4-HNE), which form adducts with proteins and nucleic acids, acting not only as cytotoxic agents but also as active signaling mediators that can modulate survival, stress response, and inflammatory pathways in tumor cells [9]. Moreover, in a recent study, it has been demonstrated that neuronal differentiation is associated with changes in mitochondrial metabolism and antioxidant defenses, further highlighting the tight interplay between redox status and NB cell fate [10].

Cells counteract aldehydic stress through the activity of NAD(P)H-dependent reductases. Among these, AKR1B1 and carbonyl reductase 1 (CBR1) play pivotal roles in the detoxification of carbonyl compounds. CBR1 catalyzes the reduction of glutathionylated 4-HNE (GS-HNE) [11], thereby conferring a context-dependent dual function in aldehyde detoxification and pro-inflammatory signaling. Conversely, AKR1B1 not only reduces GS-HNE [12] but also efficiently metabolizes free 4-HNE [13] and structurally related α,β-unsaturated aldehydes, thereby preventing aldehyde-mediated protein modification and limiting oxidative damage [14].

Despite their well-established biochemical roles, the contribution of aldehyde-detoxifying enzymes to NB response to metabolic (hyperglycemia) and oxidative (4-HNE) stress remains poorly defined. Particularly, it is unknown whether AKR1B1 and CBR1 activities function as protective mechanisms that sustain tumor cell survival under these distinct stress conditions.

In this study, we investigated the role of AKR1B1 and CBR1 in NB cells exposed to two independent stress conditions: (i) hyperglycemia-induced metabolic stress and (ii) oxidative stress induced by exogenous 4-HNE. To this end, we evaluated enzyme activities, protein expression, and cell viability both in 2D and 3D culture models, using pharmacological inhibition of AKR1B1 and CBR1.

## 2. Materials and Methods

### 2.1. Materials

Cell culture media, fetal bovine serum (FBS), non-essential amino acids (NEAAs), sodium pyruvate, penicillin/streptomycin solution, and glutamine were purchased from Euroclone (Pero, Milan, Italy). NADP^+^, NAD^+^, NADPH, NADH, and L-idose were from Biosynth (Compton, UK).

D-Glucose, D,L-dithiothreitol (DTT), isopropyl-β-D-1-thiogalactopyranoside (IPTG), Sorbinil, 3-(4,5-dimethylthiazol-2-yl)-2,5-diphenyltetrazolium bromide (MTT), phenylmethylsulfonyl fluoride (PMSF), hygromycin, phosphate-buffered saline (PBS), and Xanthine Oxidase from bovine milk were obtained from Merck Life Science (Milan, Italy). All inorganic reagents were from Avantor (Radnor, PA, USA). 4-HNE was synthesized as previously described [13].

### 2.2. Cell Cultures

Human neuroblastoma SH-SY5Y cells were obtained from ATCC (Rockville, MD, USA), and LAN-5 cells were kindly provided by Prof. M. G. Garcia. NB cells were maintained at 37 °C in a humidified atmosphere with 5% CO_2_ in a 1:1 Ham’s F-12 medium and Eagle’s Minimum Essential Medium (EMEM), supplemented with 10% (*v*/*v*) FBS, 50 mU/mL penicillin/streptomycin, and 2 mM glutamine (complete medium). EMEM was prepared from MEM supplemented with 1% (*v*/*v*) essential amino acids and 1 mM sodium pyruvate.

Cells were maintained in complete medium until reaching 80% confluence, washed twice with PBS, and subcultured using Trypsin/EDTA solution. Before treatments, NB cells were seeded at a density of 25,000 cells/cm^2^ and cultured for 72 h in complete medium, followed by 24 h in medium containing 0.5% FBS. NB cells were cultured in medium containing 0.5% FBS supplemented with D-glucose (up to 75 mM) for 24 h as an in vitro model of hyperglycemic and/or metabolic stress, a condition commonly used to activate oxidative stress pathways [15]. NB cell viability was evaluated following exposure to different concentrations of 4-HNE (from 2.5 to 20 µM) to stimulate the activation of the stress pathway, as reported elsewhere [16]. For 4-HNE treatments, cell incubation in the presence of the aldehyde was performed in FBS-free medium.

### 2.3. Assessment of Cell Viability

Cell viability was assessed using the MTT assay, according to the general procedure [17]. Briefly, prior to the analysis, complete culture medium was removed and replaced with FBS-deficient medium. Cells were then incubated for 60 min at 37 °C with 0.5 mg/mL MTT dissolved in sterile PBS. The resulting formazan crystals were solubilized in an equal volume of a 2-propanol/0.04 N HCl solution, and absorbance at 563 nm was measured using an EL808 Ultra Microplate Reader (Bio-Tek Instrument Inc., Winooski, VT, USA). Cell viability was expressed as a percentage with respect to the untreated control cells.

### 2.4. Preparation of Cell Extracts

Cells were rinsed twice with PBS supplemented with 1 mM PMSF, collected with a cell scraper, and lysed by three freezing/thawing cycles of 20 min at −20 °C followed by 60 s at 42 °C. The lysate was centrifuged at 14,000× *g* for 30 min at 4 °C, and the resulting supernatant is referred to as the cell crude extract.

### 2.5. Enzymatic Activity of Crude Cell Extract

Enzymatic activities were spectrophotometrically monitored at 37 °C using a Biochrom Libra S60 spectrophotometer (Harvard Bioscience, Inc., Holliston, MA, USA) by following the variation in absorbance at 340 nm associated with NAD(P)H oxidation (extinction coefficient at 340 nm: 6.22 mM^−1^ cm^−1^). Reaction mixtures had a final volume of 700 μL and were initiated by the addition of the substrate.

The assay mixture for AKR1B1 contained 250 mM sodium phosphate buffer pH 6.8, 380 mM ammonium sulfate, 0.47 mM EDTA, 0.18 mM NADPH, and 10 mM L-idose. Other oxidoreductase activities (AKR) were measured in 0.1 mM sodium phosphate buffer pH 7.0 containing 0.18 mM NADPH and 0.1 mM 4-HNE. The assay mixture for SORD contained 100 mM Tris-HCl pH 7.4, 0.18 NADH, and 0.4 M D-fructose. SOD activity was measured by evaluating the inhibition of the reduction of cytochrome c in a coupled system with Xanthine/Xanthine Oxidase. Briefly, the assay mixture was composed of Xanthine Oxidase 0.38 mU, Xanthine 0.03 mM, and EDTA 0.014 mM in Sodium Potassium Buffer at pH 7.8 at 25 °C. One unit of enzyme is defined as the amount of enzyme which inhibits the reduction of cytochrome c by 50%.

GST activity was evaluated as described by Peroni et al. [12]. One unit of enzyme activity (U) is defined as the amount of enzyme required for the conversion of 1 μmol/min of substrate into its product under the indicated assay conditions, and the enzymatic activities are reported as specific activity (mU/mg). The protein content of crude extracts was measured according to Bradford [18].

### 2.6. Western Blot Analysis

Aliquots of crude cell extracts containing 15 μg of proteins were diluted 4:1 in sample buffer (SB: 250 mM Tris-HCl buffer (pH 6.8) containing 10% (*w*/*v*) sodium dodecyl sulfate (SDS), 30% (*v*/*v*) glycerol, 0.05% (*w*/*v*) bromophenol blue salt, and 0.7 M β-mercaptoethanol), and then incubated at 70 °C for 10 min. Samples were loaded onto 12% TGX Stain-Free™ FastCast™ Acrylamide Gels (Bio-Rad Laboratories, Hercules, CA, USA) and electrophoresed at a constant voltage of 200 V for 45 min. Gels were exposed to UV light for 5 min using a Chemidoc Image System device (Bio-Rad) for Stain-Free gel activation. Proteins were transferred to a 0.2 μm PVDF membrane using precast Trans-Blot Turbo Transfer Pack Midi kit (Bio-Rad) with a Trans-Blot Turbo Transfer System (Bio-Rad) by applying a constant current of 1.3 A (maximum 25 V) for 6 min. Membranes were immediately analyzed with the ChemiDoc™ Imaging System (Bio-Rad) through Stain-Free Blot imaging to acquire total protein, which was used for data normalization. Total protein normalization was performed using Stain-Free technology (Bio-Rad Laboratories), which allows visualization and quantification of total protein based on tryptophan fluorescence following UV activation, according to the manufacturer’s instructions, as previously described [19]. Membranes were then treated as reported in the Supplementary Materials.

### 2.7. Sorbitol Quantification Assay

Intracellular sorbitol quantification was evaluated following the protocol performed by Felice et al. [19]. Specifically, sorbitol concentration was measured using a standard D-sorbitol calibration curve, and results were normalized to the protein content.

### 2.8. Spheroid Formation

NB cells were seeded in a F BIOFLOAT 96-well plate (FaCellitate, Mannheim, Germany) at a density of 12,500 cells/cm^2^, and spheroid formation was facilitated by centrifugation at 200× *g* for 2 min at room temperature. Cells were grown for 48 h prior to treatment. Treatments were performed as described in Section 2.2, and spheroid growth and structural integrity were monitored using the JuLI Br Live Cell Movie Analyzer (NanoEnTek USA Inc., Waltham, MA, USA). Images were taken after 24 h of incubation and analyzed using the Fiji plug-in of the ImageJ software (v1.52) [20]. Images were analyzed as described in the Supplementary Materials as reported in Figure S1.

### 2.9. Statistical Analysis

Data are presented as the mean ± standard error of mean (SEM) of the indicated number of independent experiments. Differences between two groups were evaluated using an unpaired Student’s *t*-test, whereas comparisons among multiple groups were performed using one-way ANOVA followed by the appropriate post hoc test, as indicated. The specific statistical tests applied in each analysis are reported in the corresponding figure legends. Differences with a *p*-value ≤ 0.05 were considered statistically significant. Analysis was performed using GraphPad PRISM 9 (GraphPad software, Inc., La Jolla, CA, USA) software for Windows.

## 3. Results

### 3.1. Basal Enzymatic Activity and Protein Expression of the Polyol Pathway in NB Cells

The enzymatic activity of AKR1B1 and SORD was measured in the crude extracts of the two NB cell lines (LAN-5 and SH-SY5Y) as described in Section 2.4, using L-idose and fructose as substrates, with L-idose employed as a surrogate substrate for AKR1B1 due to the higher reaction rate compared to that observed in the presence of glucose. The results are expressed in mU/mg of protein and reported in Table 1. No significant differences were observed in AKR1B1 activity between LAN-5 and SH-SY5Y cell lines. On the other hand, activity assay indicated that SORD was significantly higher in LAN-5 than in SH-SY5Y cells (*p* ≤ 0.05).

**Figure 1:**
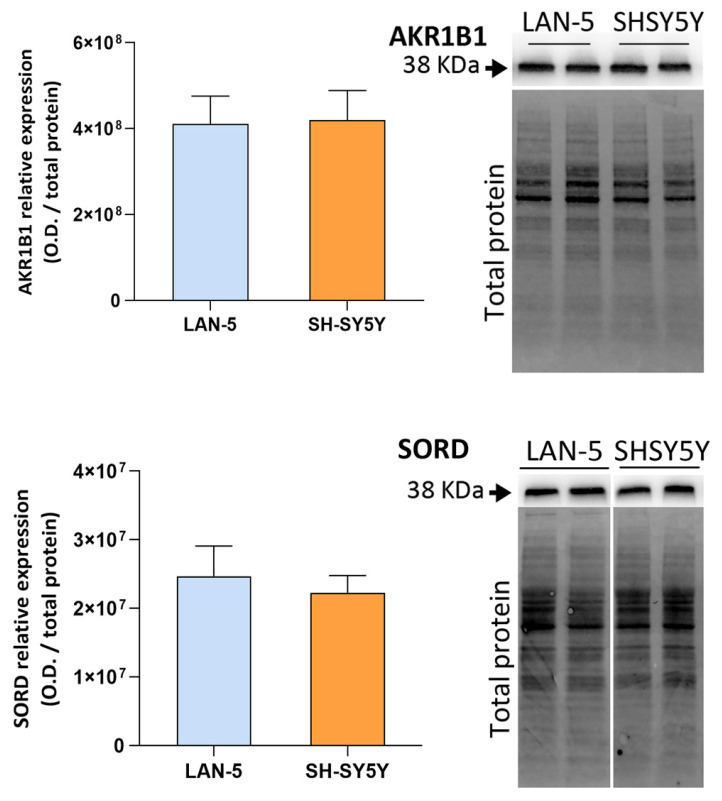
Basal protein expression of polyol pathway enzymes in LAN-5 and SH-SY5Y NB cells. The relative expression of AKR1B1 and SORD, calculated as the optical density (OD) of the proper enzyme band normalized to the total protein, is reported. Total protein level was assessed by Stain-Free technology, using Bio-Rad Image Lab software (Version 6.1.0 build 7, Standard Edition) as reported in Section 2.6. Data are reported as the mean ± SEM of 4 independent biological replicates.

When protein expression was analyzed by Western blot analysis, for both proteins, no significant differences were observed between the two cell lines (Figure 1).

### 3.2. Effect of Hyperglycemia on Polyol Pathway in NB Cells

**Figure 2:**
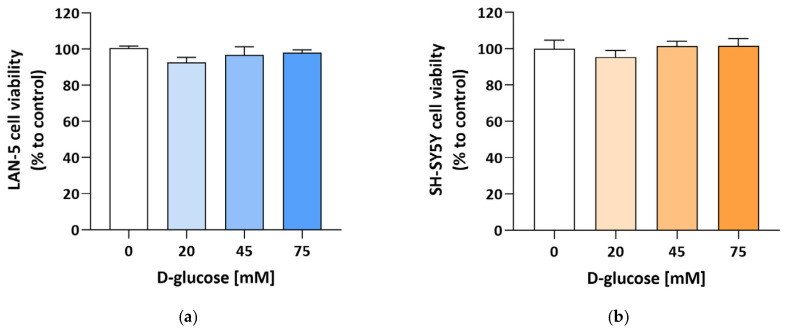
Effect of high glucose on NB cell viability. (**a**) LAN-5 and (**b**) SH-SY5Y were cultured in the presence of the indicated D-glucose concentrations added to the medium and incubated for 24 h. Data are expressed as the percentage of control cells grown in normoglycemic conditions (5.5 mM D-glucose) and are shown as the mean ± SEM of 5 independent samples. Statistical significance was assessed by one-way ANOVA followed by Tukey’s post hoc test.

The potential toxic effect of glucose was initially assessed by incubating both NB cell lines with increasing concentrations of D-glucose (up to 75 mM) and evaluating cell viability after 24 h. As shown in Figure 2, no effect was observed in either of the two cell lines.

**Figure 3:**
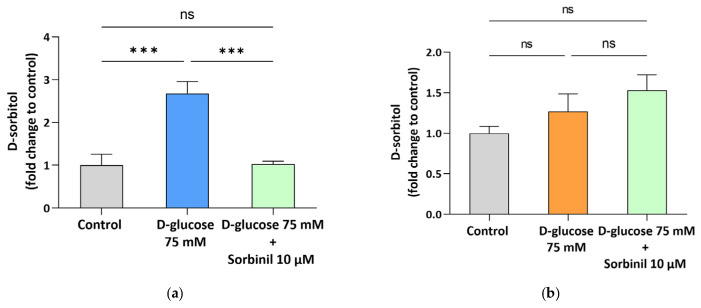
D-sorbitol accumulation in NB cell line. (**a**) LAN-5 and (**b**) SH-SY5Y were treated as indicated in Section 2.7. Data are expressed as fold change with respect to control cells grown in normoglycemic conditions (5.5 mM D-glucose) and are shown as the mean ± SEM of 3 and 5 biological independent experiments for the LAN-5 and SH-SY5Y cell lines, respectively. Statistical significance was assessed by one-way ANOVA followed by Tukey’s multiple comparisons post hoc test. *** *p* ≤ 0.001; ns *p* ≥ 0.05.

The effect of hyperglycemic conditions on the polyol pathway was evaluated by measuring intracellular D-sorbitol accumulation after exposure of LAN-5 and SH-SY5Y NB cells to 75 mM D-glucose for 24 h. As shown in Figure 3a, LAN-5 cells exhibited a significant threefold increase in sorbitol content under hyperglycemic conditions compared to controls (basal level: 60 ± 9 nmol sorbitol/mg protein). This effect was significantly prevented by treatment with 10 µM Sorbinil, an AKR1B1 inhibitor (*p* ≤ 0.001).

In contrast, SH-SY5Y cells showed higher basal sorbitol levels (86 ± 8 nmol sorbitol/mg protein) than LAN-5, but no significant further increase upon hyperglycemic exposure was observed (*p* = 0.53 vs. control) (Figure 3b).

To further characterize the polyol pathway activation under hyperglycemic conditions in the two NB cell lines, we directly assessed AKR1B1 protein expression and enzymatic activity in crude cell extracts.

**Figure 4:**
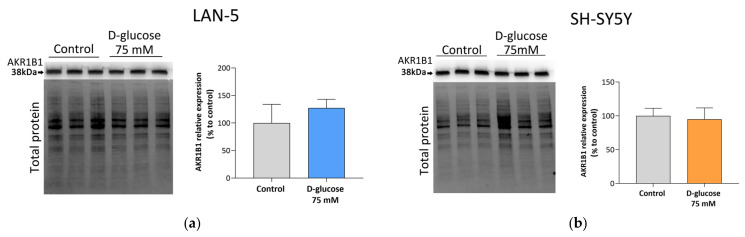
AKR1B1 expression in NB cells under hyperglycemic condition. (**a**) LAN-5 and (**b**) SH-SY5Y NB cells were incubated with or without 75 mM glucose in medium containing 0.5% FBS and harvested after 24 h. Each lane refers to a distinct biological sample (n = 3). Protein band intensities were normalized using the total protein technique as described in Section 2.6. Histograms refer to the percentage of expression of the AKR1B1 protein samples with respect to control. Statistical analysis was performed using an unpaired Student’s *t*-test (*p* = 0.5 in LAN-5 cells and *p* = 0.81 in SH-SY5Y cells).

AKR1B1 protein expression was initially assessed by Western blot in both cell lines and was not altered following exposure to 75 mM glucose in either LAN-5 or SH-SY5Y cells (*p* ≥ 0.05) (Figure 4a,b).

**Figure 5:**
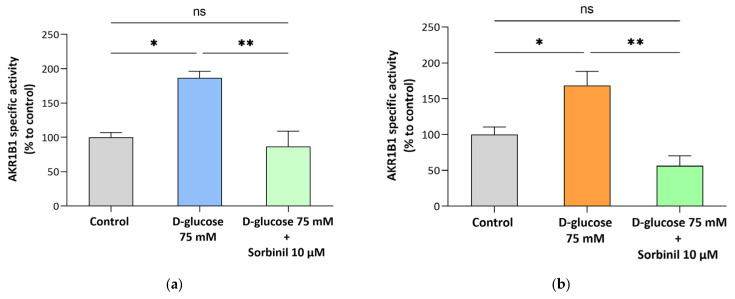
Specific activity of AKR1B1 in NB cells. (**a**) LAN-5 and (**b**) SH-SY5Y NB cells were incubated in medium containing 0.5% FBS (control) or exposed to 75 mM D-glucose, with a 24 h pre-treatment with 10 μM Sorbinil or 0.05% DMSO (vehicle control). After 24 h, cells were harvested, and AKR1B1-specific activity was measured as detailed in Section 2. Enzymatic activity is expressed as percentage to control and reported as the mean ± SEM of 3 biological independent experiments. Statistical analysis was performed by one-way ANOVA followed by Tukey’s multiple comparisons post hoc test (* *p* ≤ 0.05; ** *p* ≤ 0.01).

In contrast, despite unaltered protein expression, a significant increase in AKR1B1 enzymatic activity was detected under hyperglycemic conditions in both cell lines. Notably, the observed hyperactivation was completely reversed to control levels by a 24 h pre-treatment with Sorbinil (*p* ≤ 0.01) (Figure 5a,b). Moreover, direct comparison between the two NB cell lines revealed no significant differences in AKR1B1 enzymatic activity under any of the experimental conditions tested (Figure S2).

**Figure 6:**
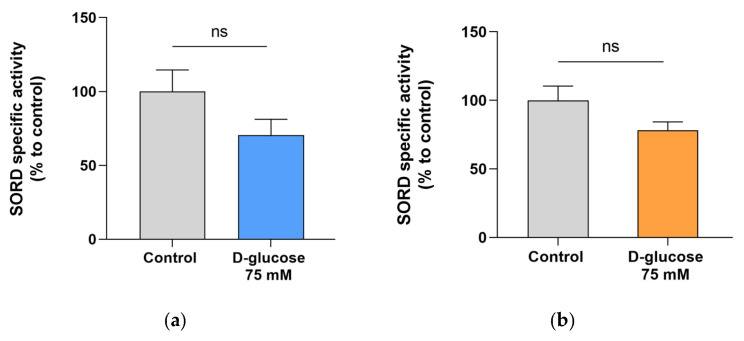
SORD-specific activity in NB cells. (**a**) LAN-5 and (**b**) SH-SY5Y NB cells were incubated under basal conditions (control) or exposed to 75 mM D-glucose. After 24 h, cells were harvested, and SORD-specific activity was measured as detailed in Section 2. Enzymatic activity is expressed as percentage to control and reported as the mean ± SEM of 4 independent biological experiments. Statistical analysis was performed using an unpaired Student’s *t*-test (*p* = 0.15 in LAN-5 cells and *p* = 0.12 in SH-SY5Y cells).

To fully evaluate the role of polyol pathway in NB cells in the presence of high glucose concentrations, SORD-specific activity was also measured. As reported in Figure 6, results revealed no significant changes under hyperglycemic conditions (*p* = 0.15 for LAN-5 and *p* = 0.12 for SH-SY5Y).

### 3.3. Antioxidant Defense System in NB Cell Lines

To evaluate the antioxidant defense system in NB cell lines, we assessed the activity of several enzymes involved in both the detoxification of reactive oxygen species and in the metabolism of lipid peroxidation products, particularly 4-HNE and its glutathionylated adduct. Oxidoreductase activities were assessed using NADPH and NADP^+^ as cofactors in the presence of various substrates, including 4-HNE, GS-HNE, and GS-NA. The A549 cell line was used as control, as AKR expression has been reported to be upregulated in this cell line [21]. Moreover, the A549 cell line was chosen as a control because it exhibits a constitutively high expression of antioxidant defense pathways [22,23].

As reported in Table 2, significant differences in the activities of antioxidant and detoxifying enzymes were observed among the three analyzed cell lines, suggesting distinct redox profiles. Superoxide dismutase (SOD) activity in NB cell lines did not show significant variations compared with the reference line; however, LAN-5 cells displayed a modest but statistically significant increase in SOD activity with respect to SH-SY5Y cells. In contrast, glutathione S-transferase (GST) activity was markedly higher in both LAN-5 and SH-SY5Y compared with A549 cells. Moreover, a significant difference was also observed between the two NB cell lines, with SH-SY5Y exhibiting the highest GST activity.

Regarding aldo-keto reductase (AKR) activities, A549 cells displayed markedly higher activity levels than LAN-5 and SH-SY5Y cells. Specifically, HNE-reductase activity was approximately 7-fold lower in LAN-5 cells and 8-fold lower in SHSY5Y cells compared with the A549 line, while AKR activity against GS-NA remained significantly higher.

Finally, we assessed the NADP^+^-dependent oxidation of GS-HNE, an activity mainly attributed to carbonyl reductase 1 (CBR1) [24]. This activity was significantly higher in A549 cells compared to LAN-5 (11 ± 2 vs. 5 ± 0.4 mU/mg, n = 3, *p* = 0.0070) and SH-SY5Y cells (11 ± 2 vs. 7 ± 1 mU/mg, n = 3, *p* = 0.0363). Additionally, a significant difference was observed between LAN-5 and SH-SY5Y cells (5 ± 0.4 vs. 7 ± 1 mU/mg, *p* = 0.0324), indicating variation in basal CBR1 activity among neuroblastoma lines.

### 3.4. Protein Expression of Enzyme Involved in HNE Detoxification

**Figure 7:**
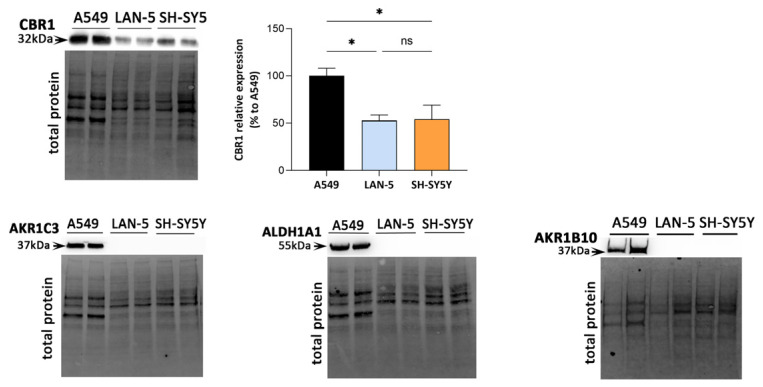
CBR1, AKR1C3, ALDH1A1, and AKR1B10 basal protein expression in NB cells. All Western blot analyses were performed on 4 biological replicates (two independent gels, each loaded with two independent cell lysates per cell line). A549 cells were included as a positive control for aldehyde-detoxifying enzyme expression. Protein band intensities were normalized using the total protein normalization method as described in Section 2.6. Bar graphs show CBR1 expression levels expressed as a percentage relative to A549 cells. Statistical analysis was performed only for CBR1 using one-way ANOVA followed by Tukey’s post hoc test (* *p* ≤ 0.05).

Although the investigated enzymatic activities are indicative of the overall detoxifying capacity of the cells, they cannot be directly attributed to specific enzymatic activities. Thus, expression of enzymes involved in 4-HNE metabolism was evaluated by Western blotting. The basal expression of enzymes known to participate in 4-HNE detoxification, including CBR1, aldehyde dehydrogenase 1 (ALDH1A1), AKR1B10, and AKR1C3, is reported in Figure 7. The A549 cell line was used as the positive reference control [21].

Among these enzymes, CBR1 was the only one expressed in all cell lines. As reported in Figure 7, a significant reduction in CBR1 protein expression was detected between NB cell lines and A549 cells (*p* ≤ 0.05), in line with the specific activity reported in Table 2. On the other hand, no detectable expression of ALDH1A1, AKR1B10, and AKR1C3 was observed.

### 3.5. Effect of HNE Treatment on Cell Viability in 2D and 3D Models

**Figure 8:**
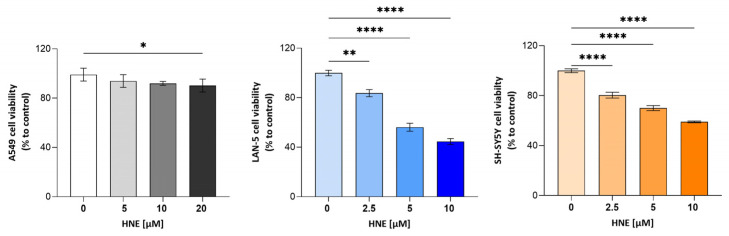
Effect of different concentrations of HNE on cell viability in A549, LAN-5, and SH-SY5Y cell lines. Cells were incubated for 24 h with the indicated concentrations of 4-HNE. Data represents 6 independent biological replicates. Statistical analysis was performed using one-way ANOVA followed by Dunnett’s post hoc test (* *p* ≤ 0.05; ** *p* ≤ 0.01, **** *p* ≤ 0.0001).

Overall, the results indicate a predominant contribution of AKR1B1 and CBR1 to reactive aldehyde detoxification in the analyzed NB cell models. To assess the sensitivity of the selected cell lines to HNE-induced cytotoxicity, cell viability was evaluated following exposure to different concentrations of 4-HNE (from 2.5 to 20 µM). As reported in Figure 8, treatment of A549 cells with 4-HNE for 24 h did not induce significant changes in cell viability at concentrations up to 10 µM, whereas a statistically significant reduction was observed at 20 µM (*p* ≤ 0.05). In contrast, both NB cell lines exhibited a marked sensitivity to HNE, with a significant reduction in cell viability observed already at the lowest concentration tested (2.5 µM).

**Figure 9:**
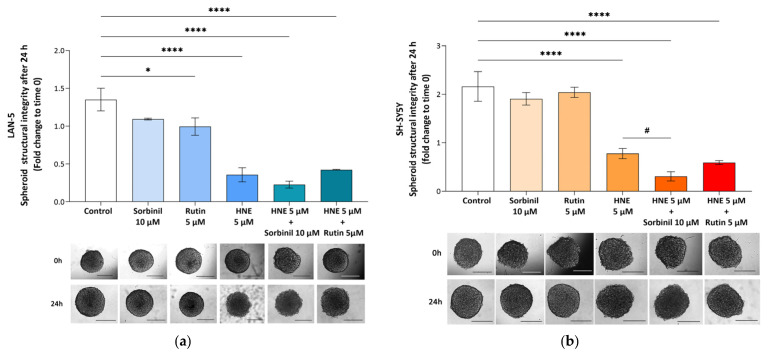
HNE-induced toxicity in NB spheroids. (**a**) LAN-5 and (**b**) SH-SY5Y NB cells were seeded in F-BIOFLOAT 96-well plates, and treatments were performed as described in Section 2. Where indicated, a 24 h pre-incubation with each inhibitor was carried out. Image analyses were performed using Fiji (a distribution of ImageJ). Images were analyzed as described above. Data are expressed as fold change with respect to time 0 and are shown as the mean ± SEM of 4 biological independent experiments. Statistical analysis was performed using one-way ANOVA followed by Dunnett’s post hoc test (* *p* ≤ 0.05; **** *p* ≤ 0.0001 vs. control; # *p* ≤ 0.05 vs. HNE). Scale bar = 250 µm.

The effect of HNE was also evaluated in a three-dimensional (3D) culture model using NB cell spheroids. The effect of 5 µM 4-HNE, which induced a 50–70% reduction in the viability of 2D cultured NB cells, was selected for the 3D experiments. To evaluate changes in spheroid growth and structural integrity induced by 4-HNE, the effect of pre-incubation with Sorbinil (10 µM), a pharmacological inhibitor of AKR1B1, and rutin (5 µM), a CBR1 inhibitor, was also analyzed. As expected, quantitative analysis revealed a significant reduction in spheroid structural integrity following 24 h exposure to 5 μM 4-HNE compared with untreated spheroids in both NB cell lines (Figure 9), confirming the HNE sensitivity observed in 2D models. Moreover, the administration of the AKR1B1 inhibitor Sorbinil or the antioxidant rutin alone did not significantly alter spheroid structure in either the LAN-5 or SH-SY5Y cell line, except for rutin, which induced a mild but significant reduction in spheroid structural integrity in LAN-5 spheroids (*p* ≤ 0.05).

As reported in Figure 9, in SH-SY5Y spheroids, pre-treatment with Sorbinil (but not with rutin) significantly exacerbated the structural alteration induced by 4-HNE (*p* ≤ 0.05). In contrast, in LAN-5 spheroids, neither inhibitor was able to produce a valuable effect on spheroid structural integrity.

## 4. Discussion

In this study, we investigated how NB cells respond to two distinct stress conditions: hyperglycemia and oxidative stress, focusing on the involvement of the polyol pathway and 4-HNE-detoxifying enzymes.

Neuroblastoma is a highly heterogeneous disease characterized by substantial biological and phenotypic variability. In this study, LAN-5 and SH-SY5Y cell lines were selected to represent distinct NB cellular contexts. LAN-5 cells are commonly used as a model of aggressive, poorly differentiated neuroblastoma, whereas SH-SY5Y cells, used here in their undifferentiated state, retain a proliferative neuroblastoma-like phenotype and are widely employed in studies investigating metabolic regulation and oxidative stress responses [25]. While these models do not capture the full heterogeneity of neuroblastoma, they provide complementary experimental systems to investigate the cellular responses analyzed in this study.

The activation of the polyol pathway under hyperglycemic conditions is a well-established mechanism in diabetic tissues, where elevated AKR1B1 activity contributes to sorbitol accumulation, osmotic imbalance, and oxidative stress. Several studies have shown that cancer cells can activate this pathway in a similar way as part of their metabolic adaptation to high glucose availability [26,27]. Accordingly, we found that both LAN-5 and SH-SY5Y increased AKR1B1 activity when exposed to high glucose, with no parallel modification in protein expression. This suggests that hyperglycemia enhances the enzyme’s catalytic efficiency rather than its expression, or post-translational regulation through the induction of conformational changes on the protein, as previously suggested in other cell types [6,28,29].

A remarkable result is that hyperglycemia induced a significant increase in sorbitol levels only in LAN-5 cells (basal level: 60 ± 9 nmol/mg protein), despite both cell lines displaying enhanced AKR1B1 activity when exposed to high glucose. The lack of sorbitol accumulation in SH-SY5Y may be attributed to their intrinsically higher basal sorbitol content (86 ± 8 nmol/mg protein), which could influence the polyol pathway. Moreover, Sorbinil did not reduce sorbitol accumulation in SH-SY5Y cell lines. The lack of a detectable reduction in sorbitol levels upon Sorbinil treatment in SH-SY5Y cells suggests that sorbitol homeostasis in this line may be influenced by factors other than AKR1B1 activity alone. This interpretation is supported by the findings of Yorek et al. [30], who showed that in NB cells, co-exposure to fructose and glucose led to higher sorbitol accumulation compared to glucose alone, highlighting the relevance of the SORD-dependent step not only as a sorbitol-removing element but also as a sorbitol-producing one.

Overall, our results indicate that NB cells do not uniformly activate the polyol pathway under hyperglycemia. Instead, they modulate its flux depending on their metabolic phenotype, suggesting that the two NB models rely on different steady-state polyol fluxes.

In the present study, we also evaluated the HNE-detoxifying capacity of NB cells. Comparison with A549 cells allowed us to better contextualize the sensitivity of NB cells to HNE, suggesting that differences in aldehyde detoxification capacity may contribute to the observed cellular responses. Although cancer cells are known to survive under chronically elevated oxidative stress [7], our enzymatic activity profiling revealed that both LAN-5 and SH-SY5Y lack several detoxifying enzymes that play crucial roles in aldehyde metabolism. The absence of ALDH1A1, AKR1C3, and AKR1B10 expression distinguishes NB cells from A549 cells, which express these enzymes and display significantly higher activities toward 4-HNE, GS-NA, and GS-HNE.

Because ALDH1A1 is a major enzyme responsible for the irreversible oxidation of 4-HNE to its corresponding acid, its absence has been associated with increased aldehyde-induced cytotoxicity in various tumor models [8]. Similarly, AKR1C3 and AKR1B10 reduce lipid-derived carbonyls and their glutathione conjugates [9]. Their absence in NB cell models indicates an incomplete aldehyde detoxification network.

This enzymatic deficiency may explain why NB cells showed strong sensitivity to 4-HNE even at low concentrations (2.5–5 μM), whereas A549 viability remained unchanged up to 20 μM. The higher GST activity observed in NB cells may contribute to the rapid formation of GS-HNE; nevertheless, NB cells are less efficient in further processing this conjugate if compared to A549. Since GS-HNE is a signaling mediator with potential pro-inflammatory and pro-survival roles [11,31], its accumulation could amplify stress responses rather than promote detoxification.

Our results indicate that NB cells possess intrinsically weak enzymatic activity on carbonylic compounds, which contributes to their vulnerability to lipid peroxidation products. However, our data showed that AKR1B1, but not CBR1, protects NB cells against 4-HNE-induced toxicity. In fact, results from both 2D and 3D culture models demonstrate that inhibition of AKR1B1 markedly exacerbates HNE-induced cytotoxicity, particularly in SH-SY5Y spheroids. This is in line with the known role of AKR1B1 in reducing free 4-HNE, GS-HNE, and related α,β-unsaturated aldehydes [13].

It should be acknowledged that genetic validation of AKR1B1 involvement (e.g., siRNA- or CRISPR-based approaches) was not performed, representing a limitation of the present study. However, the lack of detectable expression of alternative HNE-metabolizing AKR isoforms, such as AKR1C3 and AKR1B10, in our experimental models suggests that the observed effects are most likely attributable to AKR1B1 activity. Interestingly, CBR1 inhibition did not significantly modify 4-HNE toxicity in SH-SY5Y and affected only basal spheroid integrity in LAN-5. This suggests that, in NB cells, CBR1 plays a limited role in acute 4-HNE detoxification compared with AKR1B1. This is consistent with both the weak GS-HNE–oxidizing activity detected in enzymatic assays and the lower CBR1 protein expression observed in NB cells relative to A549.

Our 3D model further emphasizes the cytoprotective role of AKR1B1: spheroids treated with 4-HNE showed extensive structural disruption, and this effect was intensified upon Sorbinil co-treatment. Comparable results were obtained in conventional 2D cultures (Figure S3), where the overall trends were consistent with those observed in spheroids, although statistical significance was generally more pronounced, likely reflecting the reduced structural complexity and more homogeneous exposure to treatments in monolayer conditions. The 3D spheroid model is considered more physiologically relevant, as it better reproduces tumor architecture, diffusion gradients, and drug penetration, which may attenuate apparent drug toxicity compared with 2D systems.

Finally, assessment of downstream oxidative stress markers, including HNE–protein adducts [16], did not show significant differences between the experimental conditions in either cell line after HNE treatment [16] (Figure S4). We can suppose that under such cytotoxic settings, accumulation of HNE–protein adducts does not necessarily increase proportionally, likely reflecting impaired cellular metabolism and protein turnover.

## 5. Conclusions

The variations observed in the responses of NB cells to different stress stimuli reflect the modulable role of AKR1B1 in tumor progression. NB cells exhibit a markedly impaired aldehyde-detoxifying system and a context-dependent activation of the polyol pathway. AKR1B1 emerges as a central enzyme for managing both hyperglycemic and aldehydic stress, with its inhibition significantly reducing 4-HNE toxicity. Therapeutic strategies must therefore consider the metabolic cell context.

## Figures and Tables

**Table 1:** Basal activity of polyol pathway enzymes in NB cell lines.

Enzyme Activity (mU/mg)	LAN-5	SH-SY5Y	LAN-5 vs. SH-SY5Y (*p*-Value)
AKR1B1	8 ± 0.8	4 ± 0.8	0.059
SORD	12 ± 1	7 ± 0.7	0.012 *

Activities are expressed as milliunits per milligram of protein (mU/mg protein). Values are reported as mean ± SEM of n = 3 independent biological replicates. Statistical analysis was assessed by two-way ANOVA followed by Sidak’s multiple comparisons test (* *p* ≤ 0.05).

**Table 2:** Specific activities of antioxidant enzymes.

Enzymes	Substrate	A549 [mU/mg]	LAN-5 [mU/mg]	SH-SY5Y [mU/mg]	A549 vs. LAN-5 (*p*-Value)	A549 vs. SH-SY5Y (*p*-Value)	NB vs. NB (*p*-Value)
**SOD**	-	13 ± 3	14 ± 0.6	12 ± 1	-	-	0.0411 *
**GST**	-	133 ± 1	164 ± 2	181 ± 3	<0.0001 *	<0.0001 *	0.0012 *
**AKRs**	4-HNE	40 ± 3	6 ± 0.3	5 ± 1	0.0024 ^#^	<0.0001 *	-
**AKRs**	GS-NA	26 ± 2	13 ± 2	15 ± 3	0.0013 *	0.0062 *	-
**CBR1**	GS-HNE	11 ± 2	5 ± 0.4	7 ± 1	0.0070 *	0.0363 *	0.0324 *

SOD: superoxide dismutase; GST: glutathione S-transferase; AKR: aldo-keto reductase. Data represent 3 independent biological replicates per cell line and are reported as mean ± SEM. Statistical significance was evaluated using an unpaired Student’s *t*-test (*) or Welch’s *t*-test (#), as appropriate.

## Data Availability

The original contributions presented in this study are included in the article/Supplementary Materials. Further inquiries can be directed to the corresponding author.

## Supplementary Materials

The following supporting information can be downloaded at https://www.mdpi.com/article/10.3390/antiox15030298/s1, Figure S1: Representative image of spheroid analysis; Figure S2: Specific activity of AKR1B1 in NB cells; Figure S3: Effect of 24 h HNE treatment and enzyme inhibitor on cell viability in NB cell lines; Figure S4: HNE–protein adduct expression in NB cell lines treated with 2.5 µM 4-HNE; Table S1: Details of utilized antibodies.
