# Supplementary material for: Metabolic Detoxification of Glucose and 4-Hydroxynonenal in Human Neuroblastoma Cell Models
Source: Antioxidants (Basel). 2026 Feb 27;15(3):298. doi: 10.3390/antiox15030298 (PMC13023661; doi:10.3390/antiox15030298)
Supplement: Supplementary file 1 [file antioxidants-15-00298-s001.zip › antioxidants-4113062-supplementary.pdf]

Supplementary Materials

1. Materials and methods

1.1 Western blot analysis

Membranes were rinsed three times for 5 min in 50 mM Tris-HCl buffer pH 7.5 containing 150 mM NaCl and 0.1% (v/v) Tween® 20 (TBS-T) and blocked for 1 h at room temperature with 5% (w/v) non-fat dry milk (Cell Signaling Technology) in TBS-T (blocking buffer). Subsequently, membranes were washed three times with TBS-T for 5 min and incubated overnight at 4°C with the primary antibody.
After incubation with the primary antibody, membranes were washed three times with TBS-T and incubated for 1 h at room temperature with the appropriate horseradish peroxidase (HRP)-conjugated secondary antibody diluted 1:1000 in blocking buffer. Membranes were then washed three times with TBS-T and twice with TBS, and immunoreactive bands were detected using the Immobilon® Western Chemiluminescent HRP substrate (Merck) and captured with the ChemiDoc™ Imaging System (Bio-Rad). Membrane were blocked and incubated with primary antibodies followed by HRP-conjugated secondary antibodies. Details of the antibody, including host species and dilutions in blocking buffer are reported in Table 1.
Total protein normalization was performed using Bio-Rad stain-free technology. Briefly, gels were UV-activated prior to transfer, and total protein images were acquired using the ChemiDoc™ Imaging System before antibody incubation. Quantification of immunoreactivity bands was performed using Image Lab™ software (Bio-Rad) and band intensities were normalized to the corresponding total protein signal for each lane to correct for loading and transfer variability.

Table S1. Details of utilized antibodies.

Target	Host	Supplier	Dilution
AKR1B1	rabbit	Antibodies A40367	1:1000
AKR1B10	rabbit	OriGene TA3444167	1:1000
AKR1C3	rabbit	OriGene TA327176	1:2000
CBR1	mouse	OriGene TA346903	1:1000

ALDH1A1	rabbit	CellSignaling 54135S	1:1000
SORD	mouse	Proteintech 67625-1-Ig	1:5000

1.2 Expression and purification of human recombinant SORD

Plasmid amplification was performed in E. coli BL21 competent cells (Agilent, Santa Clara, CA, USA) using a heat-shock protocol consisting in 60 s at 42 °C, followed by immediate transfer on ice for 5 min. Cells were transferred into 10 volumes of sterile Luria-Bertani (LB) broth (10 g/L tryptone, 5 g/L yeast extract and 10 g/L NaCl), previously sterilized by autoclaving, and incubated for 1 h at 37 °C with agitation. Transformed cells were then cultured overnight at 37°C in LB broth supplemented with 30 µg/ml kanamycin. The following day, cultures were divided into aliquots and stored at -80°C.

For protein expression, cell aliquots were thawed and inoculated into sterile LB broth and cultured overnight at 37°C in agitation. Aliquots of the overnight culture were inoculated into a total of 500 ml of sterile broth (distributed into 4 Erlenmeyer flasks), and incubated at 37°C with agitation until reaching an OD_{600} of 0.7. Recombinant protein expression was induced by the addition of 0.4 mM IPTG, and cultures were incubated for an additional 3 h at 37°C. Afterwards, cells were harvested by centrifugation at 3,500xg for 15 min at 4 °C, and the pellet was resuspended (2 ml/gr) in 50 mM Tris HCl buffer pH 7.4 containing DTT 0.5 mM and PMSF 1 mM. Crude cell extracts were prepared by one freeze-thaw cycle of 20 min at -20°C followed by 1 min at 37°C, and lysates were obtained by five sonication cycles of 10 sec with 20 sec intervals on ice each. The lysate was clarified by centrifugation at 10,000xg for 90 min at 4°C, and the resulting supernatant was defined as the crude extract.

Protein purification was performed at 4°C using two sequential chromatographic steps in 50 mM sodium phosphate buffer pH 7.0 (purification buffer). In the first step, the extract was applied to a DEAE Sepharose CL-6B column (9 x 2.5 cm, Merck Life Sciences) pre-equilibrated with purification buffer containing 2 mM DTT. Proteins were eluted in the purification buffer supplemented with 2 mM DTT at a flow rate of 40 ml/h, and fractions were collected every 10 min. Protein-containing fractions were monitored by absorbance at 280 nm to estimate the protein content, assayed for enzymatic activity (as described in Section 2.9) and pooled. The pool was concentrated using YM30 Amicon ultrafiltration membrane against nitrogen pressure, and protein concentration and activity were determined by Bradford and enzymatic assays, respectively.

The concentrated protein solution was diluted 1:5 and applied to an Affinity Blue column (1.6 x 6.5 cm, Merck Life Sciences) pre-equilibrated with purification buffer. Fractions were collected every 6 min at a flow rate of 40 ml/h and assayed to confirm retention of the recombinant enzyme. After washing with 20 ml purification buffer, bound proteins were sequentially eluted with 60 ml purification buffer containing 0.37 M NaCl, followed by purification buffer supplemented with 2 mM DTT, 0.37 M NaCl and 0.1 mM NAD+. Fractions having enzymatic activity were pooled, concentrated using YM30 Amicon ultrafiltration membrane against nitrogen pressure, and analyzed for protein concentration and enzymatic activity as described before. Purification was verified by SDS-PAGE (12% acrylamide) followed by Coomassie Brilliant Blue staining. Purified recombinant SORD was divided into aliquots in 20% (v/v) glycerol and stored at -80°C. Prior to use, enzyme aliquots were extensively dialyzed at 4°C against 10 mM sodium phosphate buffer pH 7.0 using a YM10 membrane and concentrated together using two centrifugation steps at 4000xg at 4°C for 9 and 7 min respectively.

1.3 Spheroids formation

Images were calibrated to 1.794 pixels/µm, background was subtracted (rolling ball radius 100–250, light background) and converted to binary masks, as reported in **Figure S1**. Particle analysis was carried out with a size threshold of 1000–∞ μm^2 to exclude single cells and debris, with appropriate options selected to display and save outlines. All parameters were optimized for neuroblastoma spheroid analysis in our system. Statistical analysis was performed using one-way ANOVA followed by Dunnett's post hoc test (n = 4).

0h LAN-5

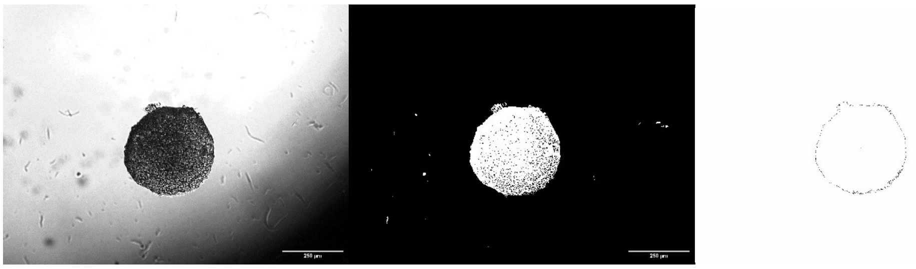

24h LAN-5

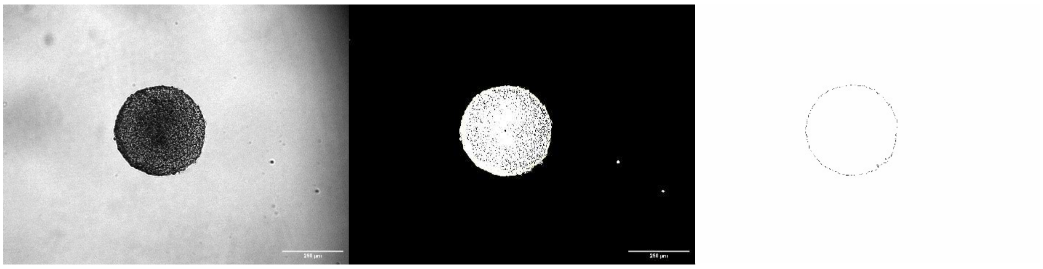

0h SH-SY5Y

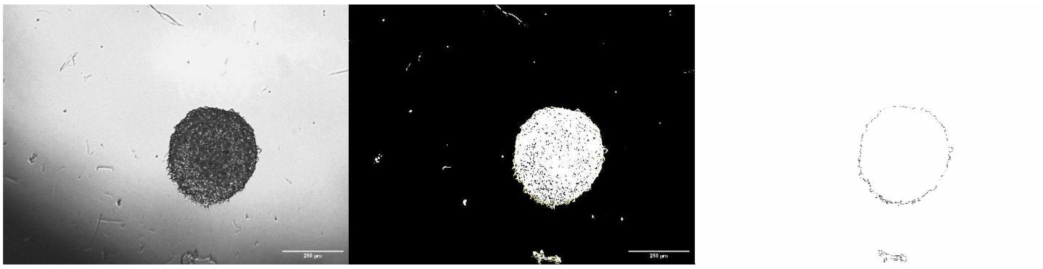

24h SH-SY5Y

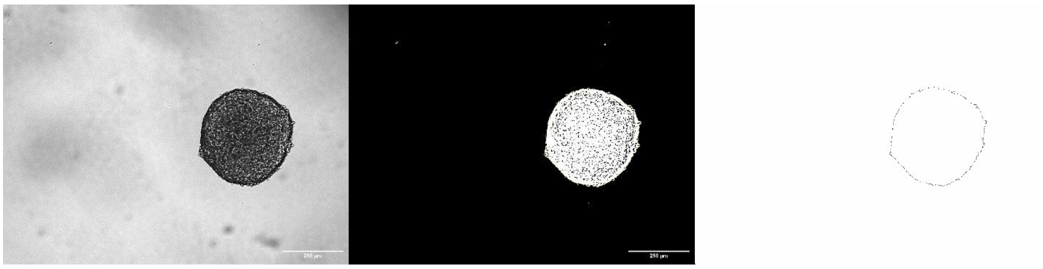

Figure S1. Representative image of Spheroids analysis. Representative bright-field (left), binary (middle), and outlined (right) images of control spheroids at 0 h and 24 h. These images show the morphological changes quantified in Figure 9 of the main text using the circularity parameter. Neuroblastoma cells (LAN-5, A and SH-SY5Y, B) were seeded in F BIOFLOAT 96-well plates, and treatments were performed as described above.

2. Results

2.1 AKR1B1 activity between cell lines under the various conditions

AKR1B1 protein activity was evaluated both in LAN-5 and SH-SY5Y NB cell lines. As reported in figure S2, no difference between the two NB cell lines has been observed.

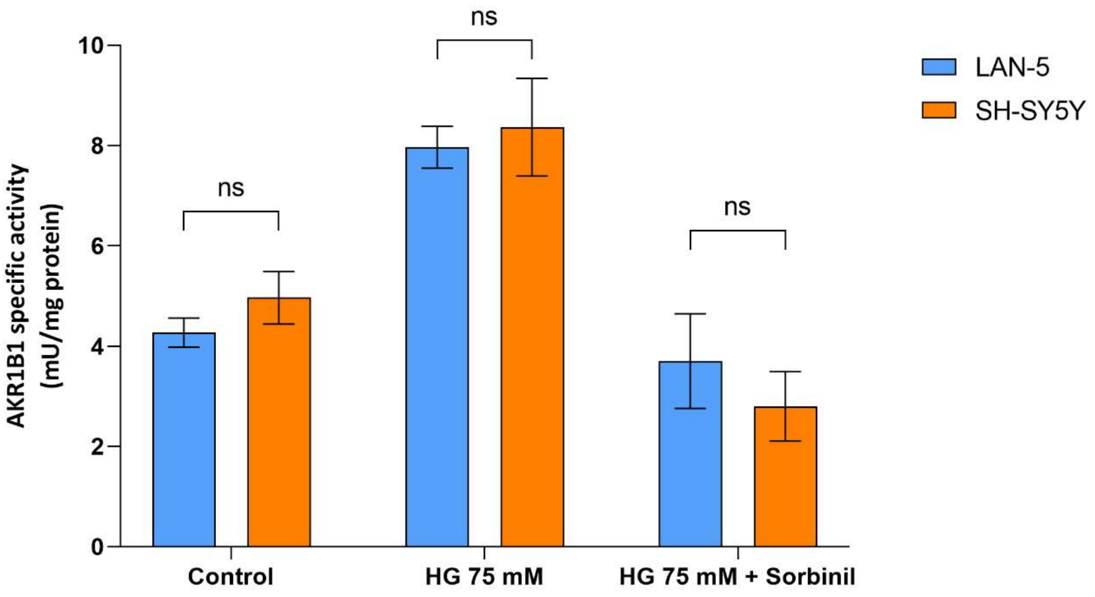

Figure S2. Specific activity of AKR1B1 in NB cells. LAN-5 and SH-SY5Y NB cells were incubated in medium containing 0.5% FBS (Control) or exposed to 75 mM D-glucose, with a 24 h pre-treatment with 10 μM Sorbinil or 0.05% DMSO (vehicle control). After 24 h, cells were harvested, and AKR1B1-specific activity was measured as detailed in Materials and Methods. Enzymatic activity is expressed as mU/mg of protein and reported as the mean ± SEM of 3 biological independent experiments. Statistical analysis was per-formed by two-way ANOVA followed by Šídák's multiple comparisons test (ns: $p \geq 0.05$).

2.2 Effect of HNE, rutin and sorbinil treatment on cell viability in 2D model

To evaluate changes in cell viability induced by 4-HNE, the effect of the pre-incubation with Sorbinil (10 μM), a pharmacological inhibitor of AKR1B1 and rutin (5 μM) as CBR1 inhibitor was also analyzed in 2D model. As reported in **Figure S3**, results revealed a significant reduction in cell viability following 24 hours exposure to 5 μM 4-HNE compared with untreated cells, in both NB cell lines ($p<0.0001$), confirming the evidence of HNE sensitiveness. Moreover, the administration of the AKR1B1 inhibitor Sorbinil did not significantly alter cell viability, in either LAN-5 or SH-SY5Y cell line. The antioxidant rutin alone, induced a significant reduction in cell viability in LAN-5 cell line ($p \leq 0.0001$) but not in SH-SY5Y.

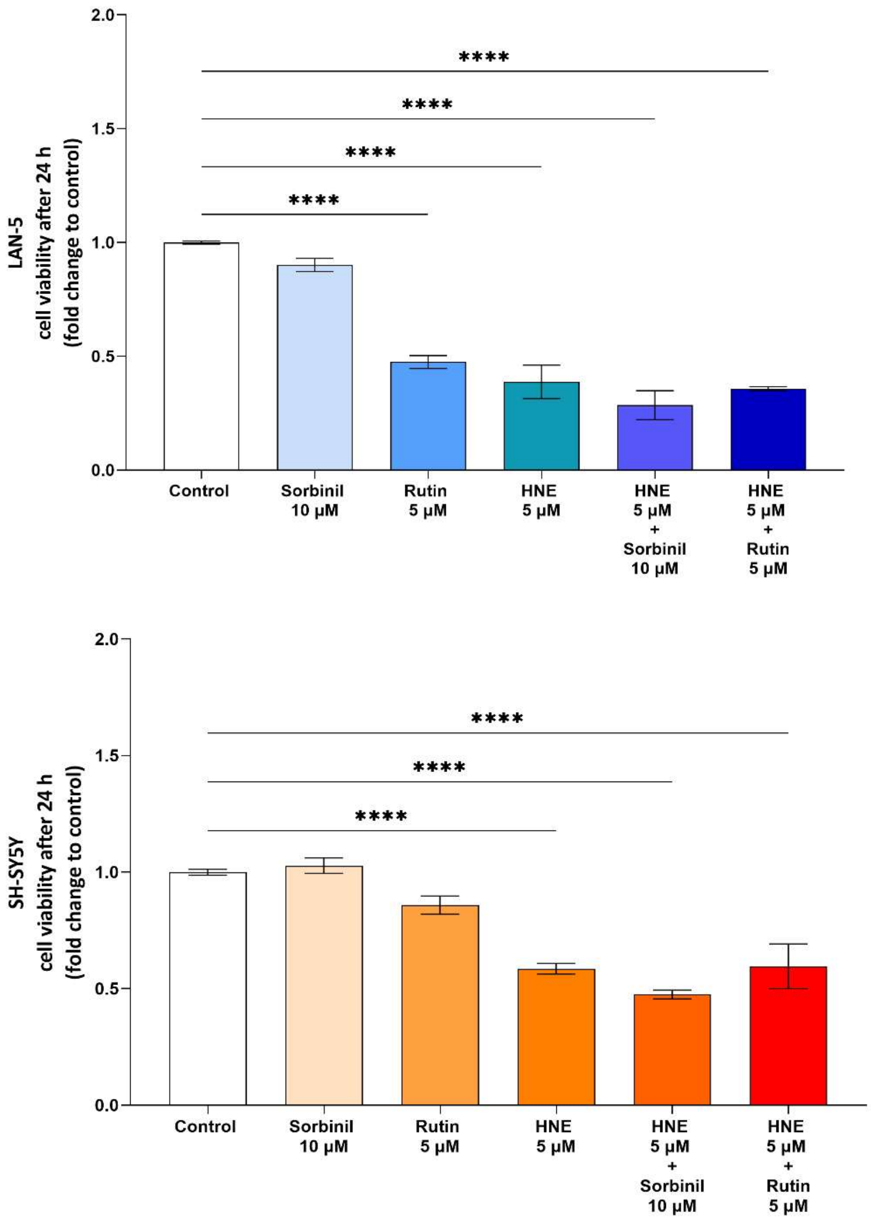

Figure S3. **Effect of 24h-HNE treatment and enzyme inhibitor on cell viability in NB cell lines.** Where indicated, a 24 h pre-incubation with each inhibitor was carried out. Cells were then incubated for 24 h with the indicated concentrations of 4-HNE. Statistical analysis was performed using one-way ANOVA followed by Dunnett's multiple comparisons test (n = 5). (****$p \leq 0.0001$ vs control)

2.3 Quantification of HNE-protein adducts by Western blot

HNE-protein adducts formation was evaluated in both LAN-5 and SH-SY5Y NB cell lines after 2.5 µM 4-HNE treatment by western blot analysis. As reported in **Figure S4**, modest HNE-protein

adducts formation was observed in both cell lines, without difference between control and HNE treatment.

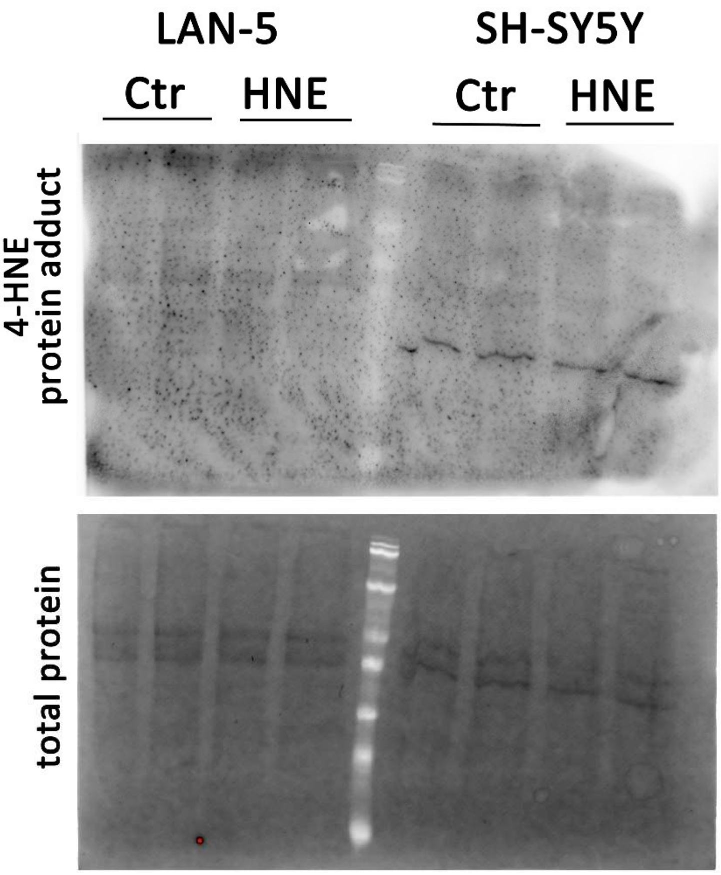

Figure S4. HNE-protein adducts expression in NB cell lines treated with 2.5 µM 4-HNE. All Western blot analyses were performed on 4 biological replicates (two independent gels, each loaded with two independent cell lysates per cell line). 4-HNE monoclonal Antibody (HNEJ-1, Cayman cat n. 38404) was used at 1:500 dilution.
